# Limb salvage surgery following resection of a melanoma: Foot and ankle reconstruction using cutaneous flaps

**DOI:** 10.3892/ol.2014.2440

**Published:** 2014-08-12

**Authors:** JIAN-FENG LIU, LI-RONG ZHAO, LAI-JIN LU, LEI CHEN, ZHI-GANG LIU, XU GONG, BIN LIU

**Affiliations:** 1 Department of Hand Surgery, First Hospital of Jilin University, Changchun, Jilin 130021, P.R. China; 2 The Electric Medical Branch, First Hospital of Jilin University, Changchun, Jilin 130021, P.R. China

**Keywords:** cutaneous flaps, foot and ankle, malignant melanoma, salvage surgery, soft tissue defects

## Abstract

Melanomas affect the foot and ankle region and are associated with a poor prognosis. The aim of the current study was to evaluate the functional and oncological outcomes of salvage surgery using cutaneous flaps for soft tissue reconstruction of the foot and ankle following the extended resection of a melanoma. A retrospective review was conducted to evaluate patients who presented with foot melanoma and underwent salvage surgery and defect reconstruction using three types of cutaneous flap (group S) or amputation (group A) between January 1999 and December 2010 at the First Hospital of Jilin University (Changchun, China). The postoperative mortality, surgical complications, functional outcomes and oncological outcomes were evaluated. Of the 21 patients, 11 were enrolled into group S and 10 were enrolled into group A. The median follow-up time of the patients was 58 months (range, 6–92 months). In group S, a reverse sural neurocutaneous island flap was used in six patients to perform the foot reconstruction, medial plantar flaps were used in four patients and lateral malleolus flaps were used in one patient. All 11 cutaneous flaps survived and provided satisfactory coverage. Only one cutaneous flap showed partial necrosis and required treatment comprising of debridement and regular changes to the wound dressing. The overall survival rate of patients was 65.0% and patients in the two groups experienced similar oncological outcomes. Salvage surgery with cutaneous flap reconstruction was found to be a reliable option for patients presenting with malignant melanoma of the foot and ankle.

## Introduction

Melanoma is a malignant tumor that is derived from melanin-producing melanocytes. Although melanoma is a rare disease, accounting for only 4% of all skin cancers, it is responsible for 80% of skin cancer-related mortalities worldwide (1). Between 3 and 15% of all cutaneous melanomas affect the foot and ankle region and are associated with a poor prognosis (2).

The treatment of malignant melanoma varies depending on the tumor characteristics (for example, the stage or site). Below-knee amputation is commonly adopted for the treatment of malignant tumors of the foot and ankle, which effectively reduces the recurrence rate, however, results in an increasing financial and psychological burden for the patients (3). With the progress of microsurgical reconstruction, limb salvage surgery using soft tissue reconstruction has recently emerged as a potential alternative for resectable malignant tumors of the extremities. The surgical excision of melanoma, with adequate margins, is the fundamental treatment that leads to recovery in the majority of cases (4). However, large, complex soft tissue defects often remain following tumor excision, which are difficult to reconstruct due to the exposure of the bones, joints and tendons. Conversely, foot melanoma remains a challenge to surgeons who are required to consider the oncologic resection whilst preserving limb function (including, walking, moving and other weight-bearing activities). Therefore, the availability of a safe, easy and reliable reconstructive option is required to repair the foot in the region of the melanoma. An ideal option would guarantee the complete removal of the tumor as well as preservation of the unique functions of the foot and ankle.

A variety of techniques, including skin grafting and local- or free-tissue transfer, have been used for the soft tissue reconstruction of the foot and ankle (5,6). However, each has their own disadvantages and may not be suitable for all patients. A cutaneous flap is a thick flap of skin whereby the underlying subcutaneous layer is supplied with blood vessels and nerves, and typically, a large quantity of subcutaneous fat. Various reconstructive flaps have been used for repairing soft tissue defects in the foot and ankle, including lateral supramalleolar, medial plantar, reverse sural neurocutaneous island, medial leg and lateral leg flaps. All of these types of cutaneous flap yield satisfactory results (7–9).

The lateral supramalleolar flap was initially described in 1988 by Masquelet *et al* (10), and is currently frequently used for covering major tissue defects of the foot and ankle. It is usually employed as a distally-based pedicle flap and has a wide range of coverage that includes the whole dorsum of the foot, the medial and lateral arches of the foot, and all of the regions of the heel (11). The medial plantar flap is one of the most versatile fasciocutaneous flaps of the foot, and provides an ideal choice for the coverage of small plantar and ankle defects, in terms of maintaining a good sensation and rapidly enabling ambulation (12,13). The medial plantar flap has a wide arc of rotation, which readily covers the plantar forefoot area. The reverse sural artery neurocutaneous island flap was originally described by Masquelet *et al* (14) in 1992 and is a distally-based flap, which is generally accompanied by the sural nerve and vascularized by the median superficial sural artery. It has been shown to be ideal for coverage of the heel and the region of the lateral malleolus of the foot, particularly for large soft tissue defects.

Although cutaneous flaps have successfully been used in the soft tissue reconstruction of the foot as a result of trauma, infection and ischemia (7), few studies have considered the results of cutaneous flap reconstruction in patients presenting with a foot melanoma. Therefore, the aim of the present study was to assess the effectiveness of three types of cutaneous flap for reconstructing soft tissue defects following melanoma resection.

## Patients and methods

### Patients and groups

A series of patients exhibiting melanoma involving the foot and ankle, from the First Hospital of Jilin University (Changchun, China) during the twelve-year period from January 1999 to December 2010, were retrospectively reviewed. A total of 21 patients (males, n=14; females, n=7) were enrolled in the present study and the mean age was 54.2 years (range, 39–70 years). The inclusion criteria were as follows: i) The patient diagnoses were validated by postoperative biopsy; and ii) the patient’s skin lesion was confined to the foot and ankle. Among the patients, 10 underwent amputation (group A) and the remaining 11 underwent salvage surgery with soft tissue reconstruction (group S). One patient was lost during the follow-up after 25 months; all of the remaining patients were followed up for an additional 6–92 months (median, 58 months) postoperatively. Patients provided written informed consent and the study was approved by the ethics committee of the First Hospital of Jilin University (Jilin, China).

### Cutaneous flap design

Cutaneous flaps were designed based on the incision size and anatomical characteristics of the affected area. Three types of cutaneous flap, including the medial plantar, reverse sural artery neurocutaneous island and the lateral supramalleolar flap, were selected to repair the soft tissue defects of the foot and ankle resulting from tumor resection. Specifically, a medial plantar flap was used for the repair of the sole and forefoot; a reverse sural neurocutaneous island flap was used for the repair of the dorsum, sole and heel of the foot; and a lateral malleolus flap was used for the repair of the lateral malleolus, dorsum and heel of the foot.

The cutaneous flap design was guided by the following rules: ii) The size and shape of the cutaneous flap must be in accordance with the defect at the recipient site (not too large or small). A cutaneous flap that is too large may cause a bloated appearance of the skin, whereas a cutaneous flap that is too small may influence the blood supply as a result of increased local tension. ii) In order to secure a sufficient blood supply, the pedicle of the cutaneous flap should be sufficiently wide and long. iii) Considering that the foot comprises thin skin, subcutaneous tissue and substantial joint activity, the cutaneous flap for foot and ankle reconstruction is required to be as thin as possible. If the cutaneous flap is too thick or bloated, it would inevitably affect the activities of the ankle joint and aesthetics of the foot. iv) The cutaneous flap for plantar reconstruction must be wear-resistant and allow a protective sensation.

### Surgical technique

Melanoma tumors were excised based on their site of origin and anatomical characteristics. In group S, lesions were widely excised using a margin of 3–5 cm in all cases and all excisions extended into or included the deep fascia. Following resection, frozen tumor sections underwent pathological testing, in order to identify clean margins, and the exact size of the soft tissue defect. Patients in group A underwent amputation, including leg amputation, tarsometatarsal amputation and toe amputation.

### Postoperative care and follow-up

Following surgery, all patients were admitted to a specialized microsurgery intensive care unit for monitoring. The patients were administered with postoperative chemotherapy and biological therapy, either alone or in combination with dacarbazine, cisplatin and intramuscular injection of interleukin-2. Surgical complications, cutaneous flap survival, tumor recurrence and metastasis, recovery of foot function, sensory recovery, and patients’ complaints were observed during the hospital stay and the follow-up period. The therapeutic efficacy was observed in each group and compared.

## Results

### Patient cohort

The demographics of the patients in each group are summarized in Table I. Among 21 patients, males outnumbered females at a ratio of 2:1 (males, n=14; females, n=7), although previous studies revealed that melanoma may be more prominent in females (15). Of the 21 patients diagnosed with melanoma, nine were of the left foot and 12 were of the right foot. In the present study, various sites of the foot were found to be affected, including the sole of the foot (n=7), the heel (n=4), the dorsum of the foot (n=3), the toe (n=5) and the ankle joint (n=2). Patients commonly complained of an increasing black mass, which bled easily when touched. The mean course of disease in these patients was 60 days. According to the European Organization for Research and Treatment of Cancer criteria (16), at diagnosis, there were 11 stage-I patients, seven stage-II patients and three stage-III patients. Three patients (one in group S and two in group A) exhibited ipsilateral inguinal lymph node metastases at diagnosis, as confirmed by intraoperative inspection of a lymph node biopsy.

### Postoperative course of the patients in groups S and A

In group S, the size of the soft tissue defects following excision of the melanoma ranged from 4×4 cm to 8×11 cm. The reverse sural neurocutaneous island flap was used in six cases, medial plantar flaps were used in four cases and a lateral malleolus flaps was used in one case, for foot reconstruction (Table II). The length of the cutaneous flaps varied from 6 to 25 cm (mean, 12.2 cm) and the width varied from 4 to 10 cm (mean, 6.6 cm). The patients were administered with routine treatment, which included elevation of the affected leg, anticoagulant agents, and anti-infection medication following surgery. During the postoperative follow-up, all cutaneous flaps survived the transfer and provided stable defect coverage, good contour, and nine out of 11 patients were able to ambulate with full weight bearing and no pain. For the recovery of sensation, all five patients with medial plantar flaps or a lateral supramalleolar flap obtained a good recovery of sensation. However, when the reverse sural island flap was used as an option for soft tissue defects of the foot, certain patients complained about a loss of sensation on the lateral aspect of the foot due to the routine sacrifice of the sural nerve during surgery, which has been reported in a previous study (17).

**Figure 1:**
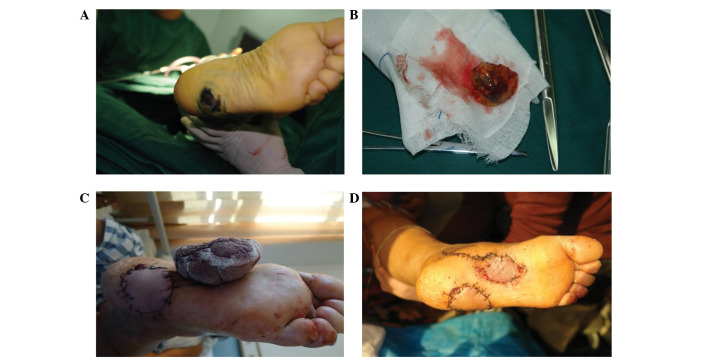
Images of female patient no. 5, aged 55 years. (A) The patient was diagnosed with a melanoma on the sole of the foot, which was identified by pathological investigation. (B) The black mass was completely removed by surgery. (C) The image was obtained three days following tumor resection and soft tissue reconstruction using a medial plantar flap. (D) Clinical image of the foot demonstrates the healed recipient site 10 days following cutaneous flap placement. The protective sensation remained functional in the affected area.

**Figure 2:**
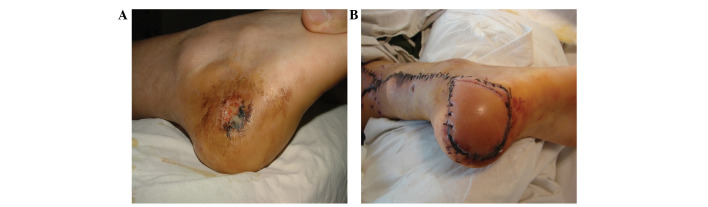
Images of female patient no. 11 (aged 50 years) who was diagnosed with a malignant melanoma on the heel of the right foot. The patient underwent defect reconstruction using a reverse sural neurocutaneous island flap following tumor resection. (A) Preoperative image. (B) The image was obtained 10 days postoperatively and demonstrates that the heel of the foot was successfully restored.

The postoperative complication rate was an important parameter to assess surgical success. The complications associated with salvage surgery using cutaneous flap reconstruction were observed in four patients. Two patients developed a mild infection at the incision site and one patient developed edema. The symptoms disappeared rapidly following symptomatic treatment. In addition to a mild infection, one of the patients developed partial necrosis at the distal tip of the cutaneous flap, however, experienced a complete recovery within two weeks following debridement and regular changing of the wound dressing (Table I). Another patient experienced a limitation of ankle plantar flexion, as the heel did not completely touch the ground when performing a squat. It was proposed that the cutaneous flap may not have been adequately loose, or the patient failed to do any exercise soon after surgery, thus resulting in scar contracture around the defects. Figs. 1 and 2 present two patients who underwent salvage surgery using cutaneous flap reconstruction (one using medial plantar flaps and the other using a reverse sural neurocutaneous island flap).

All patients in group A underwent amputation, including leg, tarsometatarsal and toe amputation. One patient was initially misdiagnosed with a benign tumor in another hospital, and thereafter underwent tumor excision surgery and skin graft. Three months later, the tumor recurred and the disease worsened. The patient subsequently attended the First Hospital of Jilin University, was diagnosed with a melanoma and underwent a lower leg amputation. Another patient, who was diagnosed with melanoma in another hospital, initially received a right toe tumor resection and defect reconstruction using a dorsalis pedis artery flap. Two weeks postoperatively, small black masses with visible exudation at the cut edges were observed on the right toe. After transfer to the First Hospital of Jilin University, pathological findings identified the recurrence of melanoma and the patient’s leg was amputated.

### Patients in groups S and A demonstrated similar oncologic outcomes

Not including one patient in group S who was lost during the follow-up period, all of the patients were followed up for 6–92 months (median, 58 months). The overall survival rate of patients was 65.0% (13/20) with a median survival time of 57 months (95% confidence interval [CI], 28.5–85.5 months) for group S and 58 months (95% CI, 50.5–65.5 months) for group A. Patients in the two groups showed similar oncological outcomes. Not including one patient in group S who succumbed as a result of cardiovascular diseases four years following surgery, none of the patients with clinical stage I (0/4 in group S and 0/6 in group A) succumbed due to melanoma-associated causes. Furthermore, no patients developed any signs of tumor recurrence or metastasis. For the seven patients with clinical stage II melanoma, excluding one patient who was lost during the follow-up period, the mortality rate in the two groups was 50% (2/4 in group S and 1/2 in group A). The three patients with clinical stage II succumbed to the disease at six, 13 and 22 months following surgery, due to tumor recurrence or inguinal/popliteal lymph node metastasis. All three patients with clinical stage III melanoma succumbed due to their disease at eight, nine and 12 months due to tumor recurrence, or pulmonary or ilioinguinal lymph node metastasis, irrespective of the group assignment (Table I).

## Discussion

Reconstruction of complex soft-tissue defects of the foot following extensive excision of a tumor remains a challenge due to the limited availability of local soft tissue, in addition to the particular structural and functional characteristics of this area. Recent studies have demonstrated and compared the benefits of cutaneous flaps for the coverage of defects of the foot and ankle (18,19), although primarily following trauma or ischemia. Few studies have investigated the benefits of salvage surgery using cutaneous flap reconstruction for the treatment of foot melanoma. The present study represents a retrospective analysis of a single-center experience for the use of cutaneous flaps for soft tissue reconstruction as part of the treatment of patients with foot and ankle melanoma.

The ideal reconstruction of the foot should provide anatomical contour, durable skin, a protective sensation and, to a certain extent, preserve limb function (standing, walking and weight-bearing activities). Commonly, the amputation of a toe does not impair the ability to walk and bear weight. Therefore, when melanoma present on a toe or on the webbing between the toes, a toe amputation at the nearest joint is often proposed prior to the disease spreading to local lymph nodes. In the present study, five patients underwent toe amputation, and one underwent leg amputation (due to inguinal/popliteal lymph node metastasis).

The type of cutaneous flap was selected based on the specific histological and anatomical features of the affected area. Skin over the sole of the foot, particularly the weight-bearing portion, requires reconstruction with similar tissues to obtain long-term function. The heel is an important, integrated aspect of the foot and is essential for smooth walking and weight-bearing activities. Without the heel, the propelling function of the foot during walking is severely interrupted. The medial plantar flap, initially described by Harrison and Morgan (20) in 1981, is a fasciocutaneous island flap raised from the non-weight bearing instep of the plantar foot. The dominant vascular pedicle of the flap consists of the medial plantar artery and venae comitantes. Furthermore, the medial plantar flap has a similar texture (thick, glabrous plantar skin, shock-absorbing fibro-fatty tissue and plantar fascia) and good sensation and is, therefore, the ideal type of cutaneous flap for plantar reconstruction, especially for weight-bearing heel defects (21). Although this type of cutaneous flap may be transferred to the defect as a proximally- or distally-pedicled island flap, it is typically used for small- or moderately-sized defects (13). Koshima *et al* (22) described a variant of this cutaneous flap that did not include fascia and was based only on a perforator of the medial plantar artery. Its advantages are that it requires an easier and shorter dissection procedure and is associated with minimal donor-site morbidity.

A reverse sural neurocutaneous island flap is presented as an alternative to the cutaneous flaps that are currently used for the reconstruction of large defects of the ankle and heel. Its anatomical structures constitute the pedicle, the superficial and deep fascias, the sural nerve, the short saphenous vein and the superficial sural artery (23). The advantages of this type of cutaneous flap include a simple dissection procedure, low donor-site morbidity and a decreased surgical time when compared with traditional coverage methods. Rohmiller *et al* (24) reported that 11 procedures using reverse sural neurocutaneous flaps have been performed for hind-foot and ankle defects (mean size, 53 cm^2^) and all cutaneous flaps achieved stable coverage. However, certain patients complained of a loss of sensation on the lateral aspect of the foot due to the routine sacrifice of the sural nerve during surgery. Dai *et al* (25) compared the clinical outcome and complications following transfer of a fascia pedicle- or a perforator pedicle-based sural neurocutaneous flap, and the results demonstrated that the latter was a more reliable and safe procedure for the coverage of soft tissue defects in the lower extremities.

Large, complex, soft-tissue defects on the dorsum of the foot are usually exposed to tendons, joints, bones, nerves and vessels as the skin is thin, which renders reconstruction more complex. The lateral supramalleolar flap is a fasciocutaneous flap that is raised from the lateral aspect of the lower leg and supplied by the perforating branch of the dorsal peroneal artery (26). A previous study indicated that the lateral supramalleolar flap is reliable and useful for coverage of the dorsum of the foot, and the medial and lateral arches of the foot, however, is not suitable for covering the weight-bearing surface of the foot (27). Hamdi and Khlifi (26) described eight children who underwent salvage surgery using the lateral supramalleolar flap for the reconstruction of skin defects of the ankle, heel and foot. All experienced satisfactory results, as no necrosis of the cutaneous flap was reported and the donor site morbidity was minimal. In the present study, the reverse sural neurocutaneous island flap was used in six patients, a medial plantar flap in four patients and a lateral malleolus flap in one patient, following the excision of melanoma lesions in the ankles and feet, which achieved good success rates.

In the present study, the cutaneous flaps varied in size according to the dimensions of the lesion resulting from extended resection of the tumor. The lesions ranged from 6×4 cm to 25×10 cm in diameter. The design of the cutaneous flaps is an important factor when considering the final cosmetic appearance and reducing complications during the postoperative period. In the current study, the majority of the cutaneous flaps had sufficient blood supply, which provided good anti-infection protection. Only two patients developed a mild infection at the incision site following the salvage surgery, which soon disappeared following the administration of anti-infection treatment. In previous studies, the most frequently described complication of a reverse sural island flap was superficial flap necrosis. Afifi *et al* (28) conducted a retrospective study using 32 consecutive reverse sural flaps for foot and ankle defects. Four patients had minor superficial loss of the cutaneous flap and four patients experienced a delayed recovery. During the follow-up of the present study, one of the patients demonstrated superficial flap necrosis at the distal tip of the cutaneous flap, and recovered completely within two weeks of debridement and regular changes of the wound dressing.

The goal of melanoma treatment is to increase the survival rate and the quality of life of cancer patients. Walsh *et al* (29) reported that, for patients with a melanoma of the foot/ankle, the overall five-year survival rate was 52%, compared with 84% for patients with a melanoma elsewhere on the lower extremities. Kang *et al* (11) reported their experience of using a distally-based island flap for soft tissue reconstruction of the foot during limb salvage surgery for 13 melanoma patients. While all 13 cutaneous flaps survived completely and provided normal weight-bearing ambulation, four patients succumbed to their disease at seven, 10, 13 and 15 months following surgery.

As foot melanoma is rare, numerous studies have combined melanoma of the foot with the hand, leg or thigh for statistical purposes, which add great variability to the survival rate of foot melanoma, particularly when including a variety of treatment options (15). The present study compared the survival rate of patients with stage I, II or III melanoma in groups of patients who underwent reconstruction or amputation. Limb salvage surgery with cutaneous flap reconstruction exhibited a similar survival rate, as well as local recurrence and tumor metastasis, when compared with amputation. Therefore, salvage surgery is recommended as a reliable method to treat patients with melanoma of the foot and ankle, particularly for the patients with stage I melanoma, enabling them to avoid the trauma associated with leg amputation and experience an improved quality of life. In addition, all patients in the current study were administered with postoperative chemotherapy and biological therapy, alone or in combination with dacarbazine, cisplatin and intramuscular injections of interleukin-2, which contributed to the efficacy of the therapy. Furthermore, a previous study hypothesized that reasonable surgical adjuvant treatment programs aided with reducing the risk of recurrence of melanoma and improved the overall survival rate (30).

In conclusion, limb salvage surgery achieves positive oncological and functional results with adjuvant treatment, particularly for patients with stage I melanoma. The medial plantar flap, reverse sural artery neurocutaneous island flap and lateral supramalleolar flap all provide effective coverage of soft tissue defects of varying sizes on the foot following the wide excision of a melanoma.

## Figures and Tables

**Table I:** Demographics of the patients in groups S and A.

A, Group S									

							Post surgery		
							
Patient no.	Gender	Age (years)	Affected foot	Location	Tumor stage	Surgical method	Follow-up (months)	Recurrence/ Metastasis	Patient status
1	Female	60	Right	Lateral plantar foot	I	Extended resection + MPF + skin graft	48	No/No	Succumbed to CVD
2	Female	49	Left	Plantar	II	Extended resection + MPF	25^a^	No/No	N/A
3	Male	63	Right	Heel	II	Extended resection + RSIF	6	Yes/Yes (Ing.)	DOD
4	Male	63	Right	Lateral dorsal foot	I	Extended resection + RSIF	77	No/No	AWD
5	Female	55	Left	Plantar	I	Extended resection + MPF	62	No/No	AWD
6	Male	39	Right	Heel	II	Tumor resection + RSIF	13	Yes/Yes (Ing./Pop.)	DOD
7	Female	54	Right	Lateral malleolus	II	Extended resection + LSMF	57	No/No	AWD
8	Male	57	Right	Dorsum of foot	I	Extended resection + RSIF	62	No/No	NED
9	Male	70	Left	Heel	III	Tumor resection + RSIF	8	No/Yes (Lung)	DOD
10	Male	61	Left	Plantar	II	Extended resection + MPF	18	No/No	NED
11	Female	50	Right	Heel	I	Extended resection + RSIF	13	No/No	NED

B, Group A									

							Post surgery		
							
Patient no.	Gender	Age (years)	Affected foot	Location	Tumor stage	Surgical method	Follow-up (months)	Recurrence/Metastasis	Patient status

12	Female	52	Right	Plantar	I	Leg amputation	92	No/No	NED
13	Male	55	Right	Medial dorsal foot	I	Leg amputation	73	No/No	NED
14	Male	58	Left	Medial malleolus	II	Skin graft, leg amputation	22	Yes/Yes (Ing./Pop.)	DOD
15	Male	46	Left	Toe	I	Toe amputation	68	No/No	NED
16	Female	45	Left	Toe	I	Toe amputation	58	No/No	NED
17	Male	59	Right	Medial plantar foot	I	Tarsometatarsal amputation	55	No/No	NED
18	Male	49	Right	Toe	I	Leg amputation	10	No/No	NED
19	Male	59	Left	Toe	III	Toe amputation + ilioinguinal lymph node dissection	12	No/Yes (Lung)	DOD
20	Male	46	Left	Second toe	III	Toe amputation	9	Yes/No	DOD
21	Male	49	Right	Plantar	II	Leg amputation	29	No/No	NED

a This patient was subsequently lost to follow-up.

CVD, cardiovascular disease; N/A, not available; MPF, medial plantar flap; RSIF, reverse sural neurocutaneous island flap; LSMF, lateral supramalleolar flap; AWD, alive with disease; NED, alive with no evidence of disease; DOD, died from disease; Ing., inguinal; Pop., popliteal.

**Table II:** Features of the cutaneous flaps observed in the patients in Group S.

Patient no.	Type of flap	Size of wound (cm × cm)	Size of flap (cm × cm)	Sensory recovery	Wear-resistant	Short-term complication
1	MPF	4×4	6×4	Yes	Yes	None
2	MPF	5×4	8×6	Yes	Yes	None
3	RSIF	6×5	15×8	No	No	Edema
4	RSIF	11×8	25×8	No	No	None
5	MPF	6×5	8×7	No	Yes	None
6	RSIF	6×8	11×8	No	Yes	None
7	LSMF	4×4	8×4	Yes	Yes	None
8	RSIF	5×4	12×6	Yes	Yes	Mild infection
9	RSIF	8×9	25×10	Yes	Yes	Mild infection, partial necrosis
10	MPF	5×4	8×6	Yes	Yes	None
11	RSIF	6×4	8×6	Yes	Yes	Limited range of motion

MPF, medial plantar flap; RSIF, reverse sural neurocutaneous island flap; LSMF, lateral supramalleolar flap.
